# Machine learning analysis of factors affecting college students’ academic performance

**DOI:** 10.3389/fpsyg.2024.1447825

**Published:** 2024-12-23

**Authors:** Jingzhao Lu, Yaju Liu, Shuo Liu, Zhuo Yan, Xiaoyu Zhao, Yi Zhang, Chongran Yang, Haoxin Zhang, Wei Su, Peihong Zhao

**Affiliations:** Department of Science and Technology, Hebei Agricultural University, Huanghua, China

**Keywords:** XGBoost, machine learning models, learning motivation, academic performance, college students

## Abstract

This study aims to explore various key factors influencing the academic performance of college students, including metacognitive awareness, learning motivation, participation in learning, environmental factors, time management, and mental health. By employing the chi-square test to identify features closely related to academic performance, this paper discussed the main influencing factors and utilized machine learning models (such as LOG, SVC, RFC, XGBoost) for prediction. Experimental results indicate that the XGBoost model performs the best in terms of recall and accuracy, providing a robust prediction for academic performance. Empirical analysis reveals that metacognitive awareness, learning motivation, and participation in learning are crucial factors influencing academic performance. Additionally, time management, environmental factors, and mental health are confirmed to have a significant impact on students’ academic achievements. Furthermore, the positive influence of professional training on academic performance is validated, contributing to the integration of theoretical knowledge and practical application, enhancing students’ overall comprehensive competence. The conclusions offer guidance for future educational management and guidance, emphasizing the importance of cultivating students’ learning motivation, improving participation in learning, and addressing time management and mental health issues, as well as recognizing the positive role of professional training.

## 1 Introduction

In recent years, the importance of cultivating professional talents and innovation capability has become increasingly recognized. In order to become high-quality professionals, individuals need to possess not only excellent academic achievements and physical fitness but also have access to excellent universities that provide high-level learning platforms to enhance their overall qualities. Consequently, there is a current emphasis on discussing how to improve students’ learning levels in the education sector. A key aspect of this discussion involves understanding and analyzing the factors that correctly influence university students’ academic performance. Through this way, a solid theoretical foundation can be established to improve management measures and enhance students’ learning quality. Previous research by scholars such as Vermunt and Donche (2017) and Korobova (2012) has shown that a majority of teachers primarily relied on subjective judgments when evaluating students’ learning performance and methods. This subjective approach lacked scientific rigor and conceptual clarity, resulting in a limited depth of understanding and analysis of students’ overall quality, ultimately leading to lower accuracy in evaluating student’ overall quality.

In China, the increased admission of students and larger classroom sizes in most universities have posed challenges for teachers in monitoring individual students’ learning progress and cognitive development (Yu et al., 2024). Consequently, a minority of students may encounter difficulties such as exam failures or grade repetition due to the struggle of keeping up with the development, and those lacking psychological resilience are more prone to drop out. These consequences have profound implications for students’ personal, societal, and national development. Therefore, research in this field is of significant importance and value. For instance, studies by Jin (2023) have emphasized the significance of predicting university students’ academic performance in a proactive manner for enhancing overall learning outcomes in higher education in China and other countries.

The academic performance of students holds an important position in the education sector. As noted by Cabrera et al. (2001), it can be evaluated through credit scores, rankings, or specific criteria to assess students’ learning conditions and proficiency levels. These assessment methods provide insights into students’ academic performance and abilities. Furthermore, predicting academic performance serves a supervisory and warning mechanism for students.

Firstly, predicting academic performance helps students adjust their learning methods and status promptly to avoid failure as much as possible. By understanding their academic performance and rankings, students can better recognize their strengths and weaknesses. Based on the assessment results, they can adjust their study plans, improve learning methods, and seek appropriate help and support. Through timely adjustments and improvements, students can enhance their learning effectiveness and better cope with academic challenges.

Secondly, predicting academic performance also has guiding significance for teachers and counselors. Teachers can modify teaching strategies and methods promptly based on students’ actual situations to meet their needs. By observing students’ performance, teachers can understand students’ learning difficulties and issues, providing personalized guidance and support. Counselors can focus more on students at risk of failure, offering timely intervention and support to help them overcome difficulties and improve academic performance.

Additionally, schools and higher education institutions can use academic performance prediction to improve education and training plans and methods. By analyzing students’ learning outcomes and performances, schools can identify areas that need improvement to enhance overall teaching quality and students’ academic achievements. They can adjust courses, allocate teaching resources, and reform assessment methods based on students’ needs, providing a better learning environment and conditions.

In summary, predicting students’ academic performance plays a crucial role in education. It not only helps students adjust their learning methods and status timely to prevent failure but also guides teachers and counselors in better teaching practices. Thus, it is an essential basis for improving education quality and enhancing students’ academic achievements.

In recent years, educational data mining has become a hot topic in academic research. As demonstrated by Koedinger et al. (2015) and Namoun and Alshanqiti (2020), data analysis, utilizing psychology and computer technology, establishes predictive models based on machine learning knowledge, collects student learning behavior data, and extracts valuable educational information. These methods provide objective suggestions for students’ learning methods, teaching strategies, and educational models in universities, contributing to improving overall student performance and making education more efficient (Fischer et al., 2020). Li et al. (2019) conducted a questionnaire survey with first-year computer science majors at a university in China. Based on the questionnaire content, the research subjects were classified into two categories-pass and fail-to explore influencing factors. The questionnaire allowed an analysis of the correlation between influencing factors and failing grades, extraction of major influencing factors, establishment of mathematical models related to failing behavior, and the proposal of targeted recommendations.

The aims of this paper are as follows: (1) compiled a survey questionnaire on metacognitive awareness, learning participation, motivation, environmental factors, time management, and mental health based on the current learning situation and literature of the university. (2) Used chi-square tests to categorize influencing factors in the survey questionnaire into major, minor, and irrelevant factors. Employed major and minor factors from the questionnaire as labels and feature values in the proposed four mathematical models to predict whether students could pass exams, warn about students’ learning effectiveness, and make early corrections. (3) Clarified the evaluation criteria for predictive models. The experimental results showed that the Support Vector Classification (SVC) model perhaps owning the highest and most stable prediction accuracy. (4) Based on the research conclusions, proposed recommendations to improve the academic performance and overall qualities of university students from the perspectives of students, teachers, and schools.

Each section of this paper is arranged as follows: the “Literature Review” section summarized related research work, the “Factor Analysis and Mathematical Model Establishment” section analyzes influencing factors and provides corresponding mathematical models. The “Experimental Analysis” section compares experimental results and evaluates experimental models, and the “Conclusion” section summarizes the paper.

## 2 Literature review

A number of studies has been conducted on student academic performance, mainly focused on students’ behaviors, influencing factors, as well as performance prediction.

Previous studies found that factors influencing students’ academic performance could be internal or external. Internal factors include learning motivation (Entwistle et al., 1971), study emotions, study behavior, health status (Gilbert and Weaver, 2010), and cultural behaviors. External factors include family situations (parental education level, occupation, etc.) and procrastination tendencies (Hooshyar et al., 2019). For instance, Zhang and Dai (2024) discussed the relationship between students’ communication willingness and learning motivation. The results shown that academic self-efficacy has a positive impact on Chinese students’ willingness to speak. In the absence of academic self-efficacy and motivation, students are more inclined to communicate with teachers and classmates in classroom teaching. Supriadi et al. (2024) discussed the relationship between mathematical reasoning, learning anxiety and learning motivation and mathematical problem-solving performance, with the help of stratified random sampling (SRS) and structural equation models (SEM). Tsai et al. (2023) used the method of constructive learning to evaluate the students’ learning motivation and learning ability. They suggested that improving students’ ability in solving evil problems is important for students to face misstatements, mixed information, conflicting values, no clear formula and intertwined problems. Yu et al. (2024) collected data of 181 first-year students from Wenzhou University (in China). A slightly degree of correlation between “scaffolding” and “internal speech” and students’ learning motivation, flexible thinking and academic achievement was observed.

In recent years, researches have attempted to use data digging and machine learning (ML) techniques to predict students’ academic performance (Hooshyar et al., 2019). Previously, Tsai et al. (2023) explored the measurement table (solving the confidence, method of solving the problem, method to avoid style and personal control) to evaluate the students’ performance in solving problems. Xu and Sun (2023) explored the relationship between physical fitness and the academic performance among primary school students, and explores the use of machine learning techniques to predict academic performance based in physical fitness. The findings confirming the association between physical fitness and academic performance. Oudman et al. (2023) assessed the correlation of explanatory variables (e.g., interest, gender and nationality) with examination scores, and their results showed that the final scores were judged by the accuracy of the exercises, rather than general clues such as interest, nationality, as well as teachers’ judgments about students’ mathematical ability. Aslam et al. (2021) used two data sets (i.e., the mathematic and Portuguese language course, as well as information about socio-economical, educational and student’s course grades data) to predicting student performance. The Synthetic Minority Over-Sampling Techniques (SMOTE) were applied to address the imbalance issues in the datasets. The accuracy of the deep neural networks (DNN) model in predicting student performance was found to be 92.4% for the Portuguese course dataset and 94.3% for the mathematics course dataset. These accuracy rates indicate that the Deep Learning (DL) models performed well in early predicting student performance. However, it’s important to note that the researchers expressed concerns about overfitting of the model.

New research variables such as course difficulty, quality of teaching resources, and school social atmosphere have also been found to be related to academic performance (Czocher, 2017, Zhang and Lian, 2024). Zhang and Lian (2024) uses single -factor inspection to analyze the impact of meta-cognitive strategies on the performance of academic behaviors. They find that gender and cultural statuses and other population statistical factors will significantly affect students’ use of read meta-data cognitive strategies. Course difficulty has a non - negligible impact on students’ academic performance. Courses with different difficulty levels require students to possess different levels of knowledge reserve, learning ability, and learning strategies. For example, according to the research of Aguhayon et al. (2023), in advanced mathematics courses, in the more difficult sections such as complex integral and differential equation parts, if students do not have solid basic knowledge and good logical thinking ability, their performance in academic performance indicators such as assignment completion and examination scores will be significantly lower than that in the less difficult parts of the course. Moreover, the setting of course difficulty is closely related to the course objectives and teaching syllabus. A reasonable difficulty gradient helps students improve their abilities step by step, while an unreasonable difficulty may lead to students’ frustration, affect their learning motivation, and further influence their academic performance (Fortier et al., 1995).

High - quality teaching resources are important factors in ensuring students’ good academic performance. Teaching resources include textbooks, teaching equipment, online learning platforms, etc. Rafiq et al. (2024) found in a survey of multiple schools that classes using textbooks with detailed content, rich examples, and timely updates had a significantly better understanding and mastery of knowledge points among students than those using old textbooks. In addition, the degree of perfection of advanced teaching equipment, such as multimedia classrooms and laboratory equipment, also affects the teaching effect. For example, in physical experiment courses, in schools with accurate and sufficient experimental equipment, students can better understand physical principles and achieve better results in experiment reports and related theoretical assessments. The functionality and resource richness of online learning platforms are also crucial. As Brnic and Greefrath (2020) indicated, online learning platforms with rich interactive functions and diverse learning materials (such as video explanations, online tests, etc.) can significantly improve students’ autonomous learning ability and academic performance.

The school social atmosphere encompasses various aspects such as teacher - student relationships and peer relationships. A good teacher - student relationship can stimulate students’ learning motivation. Li et al. (2022) pointed out that when teachers give students positive feedback and encouragement, students have higher participation in class and are more willing to take the initiative to learn. The cooperative and competitive relationships among classmates also have an impact on academic performance. In team - based cooperative projects, as Yang and Gaowei (2024) found, a positive and supportive group atmosphere among members helps with knowledge sharing and common progress, and a moderately competitive environment can prompt students to work hard to improve themselves. On the contrary, a poor social atmosphere, such as the phenomenon of school bullying, will have a negative impact on students’ psychology, thereby interfering with learning and reducing academic performance.

According to the related researches, academic performance was correlated with metacognitive awareness, learning participation, motivation, mental health, and time management, etc. However, some issues need further addressing. On one hand, predicting academic performance in a specific subject may not reflect students’ overall learning situations, presenting limitations. On the other hand, factors influencing academic performance differ among countries and universities of different levels, leading to variations in predictive results. Existing research results cannot be fully generalized and are only applicable to ordinary universities in China. Thus, determining the main factors influencing academic performance in Chinese university students and establishing a corresponding academic performance warning system are necessary in the current status.

Therefore, the aim of this study is to explore the potential correlation between questionnaire issues and academic failures. To assess the degree of correlation between variables, various analysis methods were employed, including Pearson correlation coefficient, Kendall rank correlation coefficient, and Spearman correlation coefficient. Considering the discrete and non-equal interval characteristics of variables in the questionnaire, the study chose the Pearson chi-square test as the preliminary method for factor selection.

## 3 Methodology

The primary objective of this study is to explore the potential association between questionnaire responses and academic underperformance. Various factors influencing college students’ academic performance were included, focusing particularly on learning engagement, the complexity and quantity of learning tasks, the selection of learning methods, and the impact of professional training. Previous research has established that learning engagement is a key factor affecting academic performance, reflecting the extent of student participation in classroom activities, extracurricular learning, and group discussions (Li et al., 2022; Tsai et al., 2023). To assess the degree of correlation between variables, the research employs various analytical methods, including Pearson correlation coefficient, Kendall rank correlation coefficient, and Spearman rank correlation coefficient. Considering the discrete and non-interval characteristics of the variables in the questionnaire, the study opts for the Pearson chi-square test as the initial method for factor selection.

Generally, higher levels of learning engagement correlate with improved academic performances. To illustrate the students’ active participation both inside and outside the classroom, we analyzed the frequency of material review, teacher consultations, assignment quality, and classroom attention, aiming to understand the specific impact of learning engagement on academic performance. Additionally, the quantity of learning tasks and the choice of learning methods are significant factors influencing student performance. Many students face substantial academic burdens, and their motivation is heavily influenced by the complexity and number of tasks, which can lead to frustration in achieving their academic goals. A mismatch between learning methods and students’ needs may result in insufficient understanding and mastery of the material, negatively affecting academic achievement. Thus, it is necessary to discuss the effectiveness of learning support and guidance in helping students manage academic tasks and improve their performance. Furthermore, the role of professional training, attention, and other factors are important in fostering students’ enthusiasm for learning and enhancing their academic performance.

### 3.1 Chi-square test

In previous studies, Zander et al. (2020) discovered a correlation between effective mastery and cognition with self-efficacy in both girls and boys using the chi-square test in October 2020. Danxing (2019) similarly analyzed the correlation between classroom accumulation, pre-final exam reinforcement, and the final grades of college students using the chi-square test in 2019.

This study utilizes the Pearson chi-square test to determine the relationship between various questionnaire features and academic underperformance. Specifically, it investigates whether the variables in the questionnaire are correlated with variables indicating academic underperformance, as this relationship will directly impact the input of the predictive model. The specific steps include.

#### 3.1.1 Hypothesis testing

Assume H0 that the distributions of the two variables are not identical.

#### 3.1.2 Cross-tabulation and expected frequency calculation

Calculate the cross-tabulation of the two variables (as shown in Table 1) and the expected frequencies (as shown in Equation 1), where *T_ij_* represents the expected frequency.


(1) $$T_{ij}=\frac{\left(\sum_{j=0}^{1}x_{ij}\right)\left(\sum_{i=A}^{E}x_{ij}\right)}{\sum_{i=A}^{E}\sum_{j=0}^{1}x_{ij}}$$

**Table 1:** Cross-tab.

	0	1	Sum
A	X_A0_	X_A1_	X_A0_ + X_A1_
B	X_B0_	X_B1_	X_B0_ + X_B1_
C	X_C0_	X_C1_	X_C0_ + X_C1_
D	X_D0_	X_D1_	X_D0_ + X_D1_
E	X_E0_	X_E1_	X_E0_ + X_E1_
Sum	X_A0_ + … + X_E0_	X_A1_ + … + X_E1_	X_A0_ + … + X_E0_ + X_A1_ + … + X_E1_

#### 3.1.3 Sample size and threshold check

If the sample size (n) is greater than 40 and the expected frequency (*T_ij_*) is greater than 5, proceed to the next step; otherwise, consider using a continuity-corrected chi-square test or Fisher’s exact test. In this study, the conditions are met, so there is no need for the introduction of continuity-corrected chi-square test and Fisher’s exact test.

#### 3.1.4 Calculation of *p*-value and test statistic

Calculate the *p*-value based on the cross-tabulation, representing the probability of the two variables having disparate distributions. The calculation of the chi-square test statistic (χ2) is as shown in Equation 2.


(2) $$\chi^{2}=\overset{E}{\underset{i=A}{\sum }}\overset{1}{\underset{j=0}{\sum }}\frac{{\left(x_{ij}-T_{ij}\right)}^{2}}{T_{ij}}$$

#### 3.1.5 Check for statistical significance

Check the critical value table to observe whether the chi-square test is statistically significant. If the chi-square statistic is large enough (or the p-value is small enough), it indicates a significant correlation between the two variables (Zander et al., 2020).

*X_ij_* represents the frequency of students choosing option *i* under condition j. Here, *i* = A, B, C, D, E represents the options in the questionnaire, and *j* = 0, 1 represents all students who passed the exam, as opposed to all students who did not pass the final exam.

## 4 Mathematical model

The machine learning models used in this study are logistic regression (LOG) model, random forest classifier (RFC) model and XGBoost model.

The LOG model is a supervised machine learning algorithm commonly used for binary classification prediction tasks. In this model, the independent variable is a random real number, and the output value is between [0, 1]. The core idea of this model is that a predicted value is first calculated by linear regression, and then the predicted value is mapped by S-type function to realize the process of converting the predicted value into probability value. By default, the model uses 0.5 as the classification threshold. If the calculated probability value (usually expressed as P) is greater than 0 but less than or equal to 0.5, the sample will be classified as 0; if P is greater than 0.5 but less than or equal to 1, the sample will be classified as 1. This threshold can be adjusted according to the specific needs and the actual situation to change the classification results. In general, the LOG model is simple and efficient, but sometimes its prediction results may not be satisfactory.

The RFC model is an ensemble learning method based on decision trees in machine learning (Chen et al., 2021). It is usually used in classification and prediction tasks to construct multiple decision trees as independent classifiers, and finally produce the final classification results based on the majority voting principle. This helps improve classification performance. RFC model has advantages in dealing with high-dimensional data, because it does not need dimension reduction operation and supports parallel computing, so it improves the computational efficiency. However, in the case of small samples or low-dimensional data, the performance may be limited, which may easily lead to over-fitting or unstable results. Therefore, when choosing machine learning algorithms, we need to carefully consider the characteristics of data and the needs of the problem, and try different methods to achieve better performance.

The XGBoost model is a powerful gradient boosting machine learning algorithm for classification and regression tasks (Miguéis et al., 2018). It performs well in dealing with high-dimensional and large-scale data, supports automatic feature selection and processing missing data, and has the advantages of parallel computing. Unlike random forests, XGBoost allows optimization of the weight of each tree, with built-in regularization to prevent overfitting. This makes XGBoost the first choice for many data scientists and machine learning practitioners.

### 4.1 Model evaluation

In this study, the model evaluation was carried out for a binary classification problem, and the confusion matrix (Table 2) was used as the evaluation tool, which mainly included key indicators such as accuracy, precision, recall and ROC curve (Equations 3–5).

**Table 2:** Confusion matrix.

True value	Predict value	
	P′	N′
P	TP	FN
N	FP	TN

#### 4.1.1 Accuracy

Accuracy represents the percentage of the total number of samples correctly predicted by the model to the total number of samples.

TP: The number of samples where both the predicted value and the true value are 1;
TN: The number of samples for which both the predicted and true values are 0;
FP: The number of samples with a true value of 0 and a predicted value of 1;
FN: The number of samples with a true value of 1 and a predicted value of 0.

(3) $$\mathrm{Accuracy}=\frac{TP+TN}{TP+TN+FN+FP}$$

#### 4.1.2 Precision

Accuracy represents the proportion of samples predicted by the model to be 1, which is actually 1. Due to the small proportion of failed students in recording, the accuracy and precision are difficult to fully reflect the model’s effectiveness, so a recall rate is introduced.


(4) $$\mathrm{Precision}=\frac{TP}{TP+FP}$$

#### 4.1.3 Recall

Recall measures the proportion of samples that are successfully identified as 1 by the model among all samples that are actually 1. In the face of rare failure cases, recall provides a more comprehensive and sensitive performance evaluation, focusing on the model’s ability to identify positive examples.

Due to the relative rarity of failed student records, the accuracy and precision of the two evaluation indicators are difficult to fully reflect the performance of the model. Therefore, in order to evaluate the performance of the model more accurately, it is necessary to introduce the recall, which is also known as the recall, and pays more attention to the ability of the model to identify positive examples.


(5) $$\mathrm{Recall}=\frac{TP}{TP+FN}$$

#### 4.1.4 ROC curves

The ROC curve takes the true positive probability as the ordinate and the false positive probability as the abscissa, in the performance prediction, the ordinate represents the probability of student failure, and the abscissa represents the probability that student failure is incorrectly predicted. The closer the curve is to the vertical coordinates, the higher the prediction accuracy and the better the effect of the model.

## 5 Experimental anaylsis

### 5.1 Source of data

According to the results of previous studies and the actual situation of Chinese college students, the designed questionnaire mainly includes metacognitive awareness, learning participation, motivation, environmental factors, time management and mental health, as shown in Tables 3–8. The data used in this article come from students in the School of Science and Technology, Hebei Agricultural University. A number of 1,701 questionnaires were collected. The participants included students from their first to fourth year, primarily majoring in five majors: Environmental Science, Food Science, Computer Science, Chemical Engineering and Pharmacy, and Water Resources and Hydropower Engineering. The students mainly had a background in science and engineering, and both male and female students were randomly selected.

**Table 3:** Questions about metacognitive awareness.

Number	Question
Q1	Which of the following study techniques do you typically use when planning your studies?
Q2	Do you reflect and summarize your learning?
Q3	Will you have learning goals and plans?
Q4	Are you good at using feedback to make learning adjustments?
Q5	Are you satisfied with your college entrance exam results?

**Table 4:** Questions about environmental factors.

Number	Question
Q1	How do you feel about the learning environment at school?
Q2	Which of the following factors do you think has had a greater impact on your learning environment?
Q3	Do you feel distracted by noise in your learning environment?
Q4	How do you think relationships on campus have influenced your studies?
Q5	Do you think that the learning environment is important for the development of learning ability?
Q6	What factors in your learning environment do you think have had the greatest impact on your learning outcomes?
Q7	What is your parent’s maximum education?
Q8	What do you think of the learning atmosphere in your dormitory?

**Table 5:** Questions about learning engagement.

Number	Question
Q1	Are you actively involved in discussions and asking questions in class?
Q2	Do you actively seek out and participate in extracurricular learning opportunities such as seminars, lectures, hands-on activities, etc.?
Q3	Are you actively involved in collaborating and contributing in group projects or team assignments?
Q4	Are you willing to share learning resources, notes, or learning experiences with your classmates?
Q5	Are you involved in a learning-related organization such as a learning group, academic society or club?
Q6	In terms of course selection, do you prefer courses that involve practical application and practice?
Q7	Are you actively involved in learning-related volunteer activities or community service?
Q8	How often do you review after class?

**Table 6:** Questions about time management.

Number	Question
Q1	How do you usually organize your study time each day?
Q2	Do you often use time management tools (alarm clocks, phones, etc.) or apps (cloud study rooms, etc.) to help you plan and track tasks?
Q3	Do you often feel like you are running out of time to complete all your tasks?
Q4	Are you constantly affected by procrastination and wasted time?
Q5	Do you regularly evaluate and adjust your time management strategy?
Q6	Do you maintain a regular sleep schedule?

**Table 7:** Questions about motivation to learn.

Number	Question
Q1	Are you happy with your current state of study?
Q2	What are some of the following reasons why you typically feel less motivated to study?
Q3	What do you think is what motivates you to learn?
Q4	How do you typically cope with learning difficulties and setbacks?
Q5	What are your plans for the future?

**Table 8:** Questions about mental health.

Number	Question
Q1	How do you think your own mental health is?
Q2	What are some of the reasons why you usually feel stressed?
Q3	How do you typically deal with psychological stress?
Q4	What do you think college students should do to maintain their mental health?

To maintain a balance among the unique characteristics of different majors, the questionnaire was designed to measure multiple dimensions including training programs and curriculum design. The main focus was on the failure rates of core and foundational courses in these majors and their influencing factors. For instance, in the Environmental Engineering major, we examined courses, including Environmental Engineering, Air Pollution Control, and Water Resource Optimization; in the Food Science major, we looked at courses such as Food Chemistry, Food Microbiology, and Food Toxicology; in the Chemical Engineering and Pharmacy major, we considered courses like Pharmacy, Physical Chemistry, Drug Analysis, and Drug Separation Engineering; in the Water Resources and Hydropower Engineering major, we included courses such as Engineering Surveying, Mechanics of Materials, and Engineering Hydrology; and in the Computer Science major, we examined courses including Computer Organization Principles, Compiler Principles, Software Engineering. The relevant course design files come from the training programs of the College of Science and Engineering at Hebei Agricultural University from 2017 and 2021,^1^ while other basic information come from data in the university’s digital system database. It is important to note that general education courses like College English, College Mathematics, and Moral Education were excluded. Additionally, the study also does not address the influencing factors of elective and expanded courses, nor the complexities of graduation projects and internships related to employment and further education.

After collecting the questionnaire, we found that part of the content was invalid. Therefore, we excluded this group of students from original samples. Finally, we obtained 1,101 valid samples. The results of 1,101 freshmen in each discipline were obtained through the educational management system. We use 0 to denote all passes and 1 to denote all failures. Some of these data sets are shown in Table 9.

**Table 9:** Partial datasets.

Name	Q1	Q2	Q3	Q4	Q5	…	Q32	Q33	Q34	Q35	Q36	Pass/Fail
Huang KeHua	AC	A	A	B	C	…	A	B	A	A	ABCD	0
Zhang ZiXuan	ACD	B	B	B	C	…	D	B	ACD	BD	ABCD	1
Song YuMing	AB	B	A	A	A	…	A	A	CD	BD	ABCD	1
Wang ShiTong	AC	A	A	A	B	…	A	A	AC	AB	ABCD	0
Kong XiangXin	AC	B	B	A	C	…	A	B	AC	AB	ABCD	0

**Figure 1:**
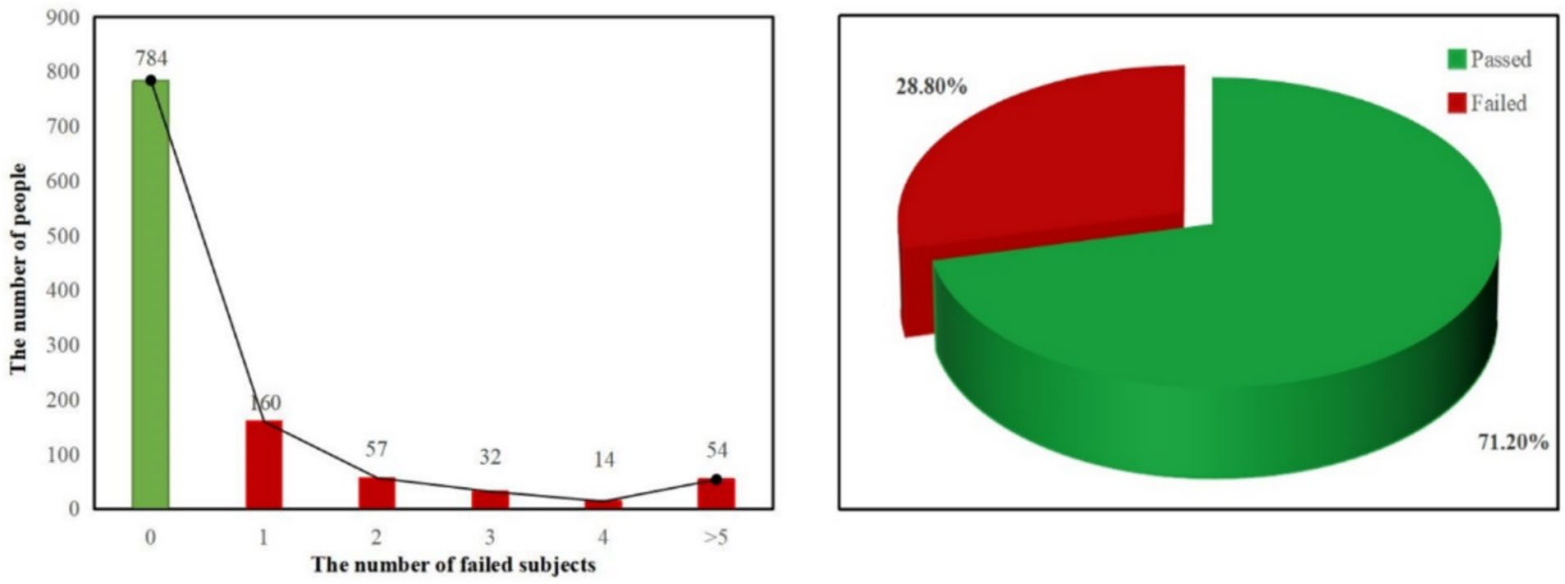
Overall academic performance.

In order to determine the academic situation of the subjects, we standardized their overall learning performance. From Figure 1, we find that 784 students passed all final exams and 160 students failed at least 1 course, accounting for 71.2 and 28.8% of the total, respectively. Among the students who failed, the number of students who failed in two examinations was 57, 32 in three, 14 in four and 54 in more than five. Overall, about a third of the students received failing grades.

### 5.2 Chi-square test results

Using the chi-square test, we successfully selected the characteristics closely related to student failure as the independent variable, and the failure record as the dependent variable. In Tables 10, 11, the results show the cross-labeling of Q1-Q5 and the corresponding expected frequency calculations. Since the number of samples *n* = 1,101 is greater than 40 and the value of T is greater than 5, the Pearson’s chi-square test was selected for further analysis. 

**Table 10:** Q1-Q5 Cross-labeling statistics for questionnaires.

Question	Q1		Q2		Q3		Q4		Q5	
Option	0	1	0	1	0	1	0	1	0	1
A	594	201	256	75	266	93	569	201	77	39
B	459	154	432	190	408	179	197	110	320	160
C	515	203	76	42	86	32	12	13	318	90
D	399	132	5	6	10	8	-	-	56	24
E	-	-	9	11	8	12	-	-	7	11

**Table 11:** Expected frequency.

Question	Q1		Q2		Q3		Q4		Q5	
Option	0	1	0	1	0	1	0	1	0	1
A	588.55	206.45	233.68	98.82	253.45	105.55	543.61	226.39	81.89	34.11
B	453.81	159.19	439.13	185.7	414.42	172.58	216.74	90.26	338.87	141.13
C	531.54	186.46	83.31	35.23	83.31	34.69	17.65	7.35	288.04	119.96
D	393.10	137.90	7.77	3.28	12.71	5.29	–	–	56.48	23.52
E	–	–	14.12	3.28	14.12	3.53	–	–	12.71	5.29

The results of chi-square test showed that the *p*-values of Q1, Q2, Q6, Q11-Q13, Q15, Q15, Q18, Q20, Q22-Q24, and Q27 were less than 0. 01, which indicated that there was a strong correlation between them and failure records, and they were identified as the main influencing factors (Tables 10, 11). The correlation of Q4, Q5, Q16, Q19, and Q26 is in the range of 0.01 < *p* < 0.05, indicating that they are more common in correlation and belong to secondary factors. As for the remaining features, p-values greater than 0.05 indicate that there is no significant correlation between them and failure records. Due to the large dimension of the data, only parts of the features Q1-Q5 are given in Tables 10, 11.

Based on the results of chi-square test, it is concluded that the factors affecting students’ academic performance in our school mainly involve metacognitive awareness, learning motivation, learning participation, environmental factors, time management and mental health. Metacognitive awareness includes factors such as college entrance examination scores, satisfaction and self-achievement. Learning motivation covers learning interest and other aspects. Learning engagement involves the extent to which students participate in classroom activities, extracurricular learning, and group discussions. High learning engagement is generally associated with better academic performance. Time management includes classroom learning, autonomous learning and the rational allocation of leisure time. Environmental factors include home learning environment, library resources, laboratory facilities and so on. Mental health includes factors such as psychological stress, anxiety and depression.

### 5.3 Model prediction results

During the modeling process, a number of labels were used to indicate whether the individual students failed the final exam, where the value is 1 or 0. The characteristics of these labels are derived from the question in the questionnaire, which is the main factor affecting students’ academic performance, and are verified by chi-square test. We took the feature columns of the top six columns ranked by the chi-square test as the feature columns at the time of our prediction.

Usually, the original data is divided into a training set and a test set before the model is trained. However, due to the small data set containing failure cases, the traditional data separation method may lead to overfitting or underfitting of the model. Usually, in order to balance the number of positive and negative samples, the over-sampling and under-sampling methods were applied to increase the number of samples, but these methods have some limitations.

**Figure 2:**
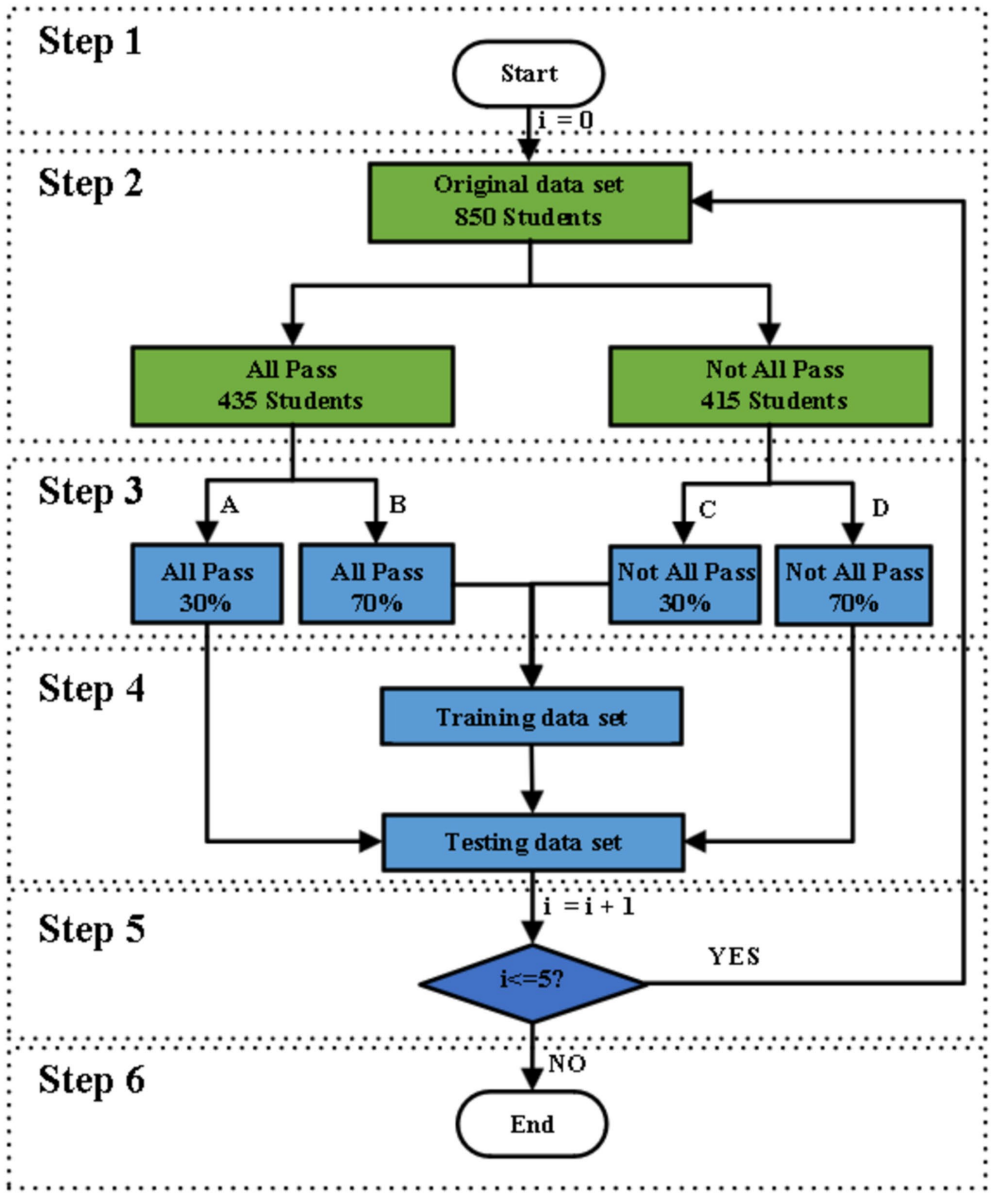
Dataset segmentation flow chart.

To overcome these problems, we introduce the idea of stratified sampling to separate the training set and the test set of the model. As shown in Figure 2, the specific process is as follows:

Step 1: Let the number of cycles be I and initialize I to 0.
Step 2: The original data set was divided into an all-pass group (784 students) and a not-all-pass group (317 students).
Step 3: Randomly assign the all-pass component to group A (30% of the data) and group B (70% of the data), and the not-all-pass component to group C (70% of data) and group D (30% of data).
Step 4: The training data set consists of the combination of group B and group C, and the test data set consists of the combination of group A and group D. Step 5: If the number of cycles I < = 5, return to step 2; otherwise, go to the next step.
Step 6: End.

In the selection of machine learning (ML) models, we use binary classification models such as LOG, SVC, RFC and XGBoost. The models are implemented using the “scikit-learn” library in Python. The model training phase is carried out by separating the training set and the test set, and the performance of the model is verified.

In the training of the XGBoost model, the parameter ***C*** and kernel were adjusted, and the best parameters with ***C*** = 1.0 and kernel = “linear” were determined by grid search. This is because a large value of ***C*** penalizes the wrong sample, but too large may lead to overfitting, while a small value of ***C*** allows a certain degree of misclassification and helps generalization. In addition, the linear kernel is considered to perform better on this problem. In terms of model evaluation, we comprehensively evaluate the model through confusion matrix, recall, accuracy and precision values. Separately, by plotting *ROC* curves, we assessed the effectiveness of the model in predicting student academic achievement. The XGBoost model performs best in both recall and precision, reaching 86.52 and 89.22%, respectively.

**Figure 3:**
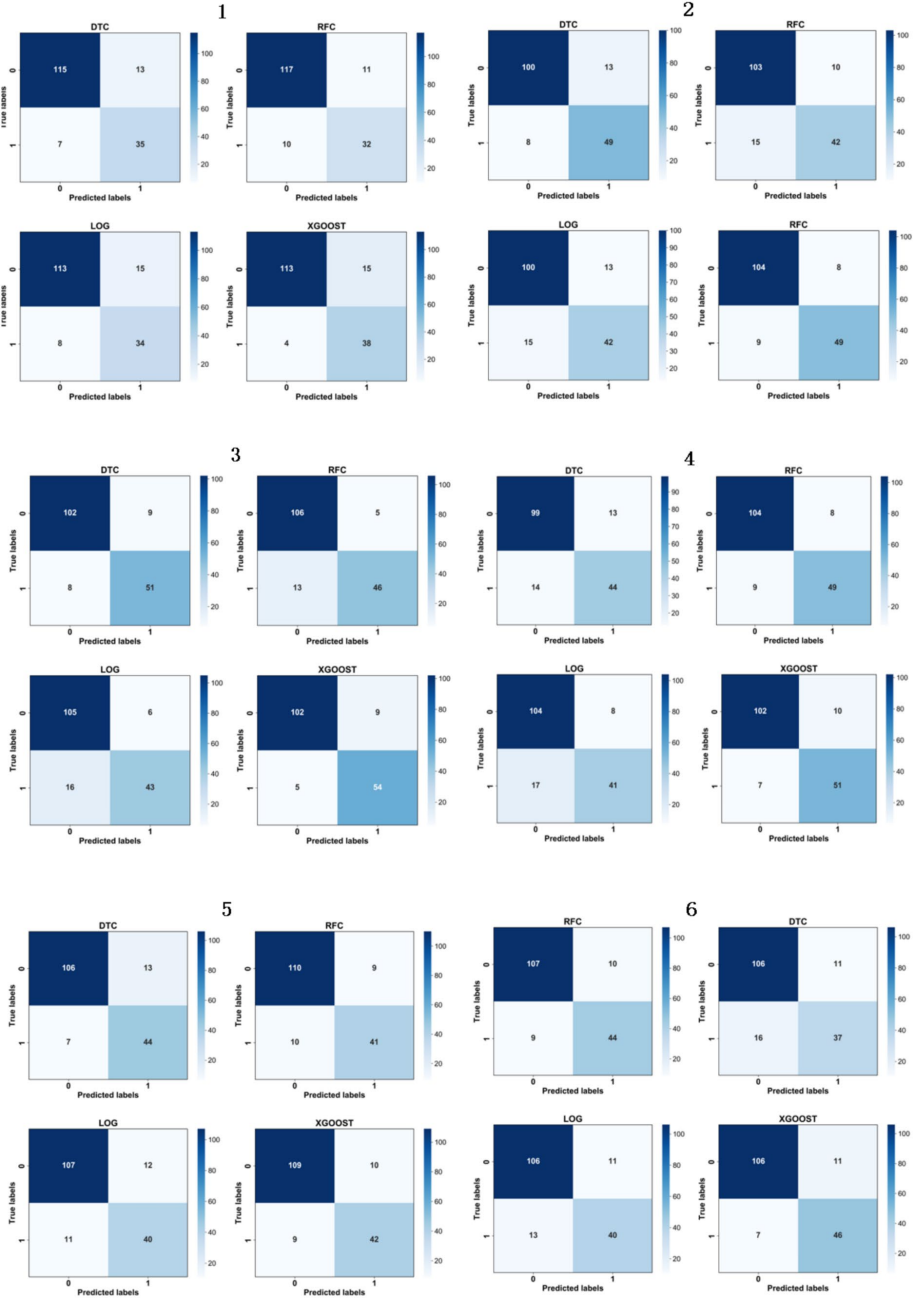
Confusion matrix.

**Figure 4:**
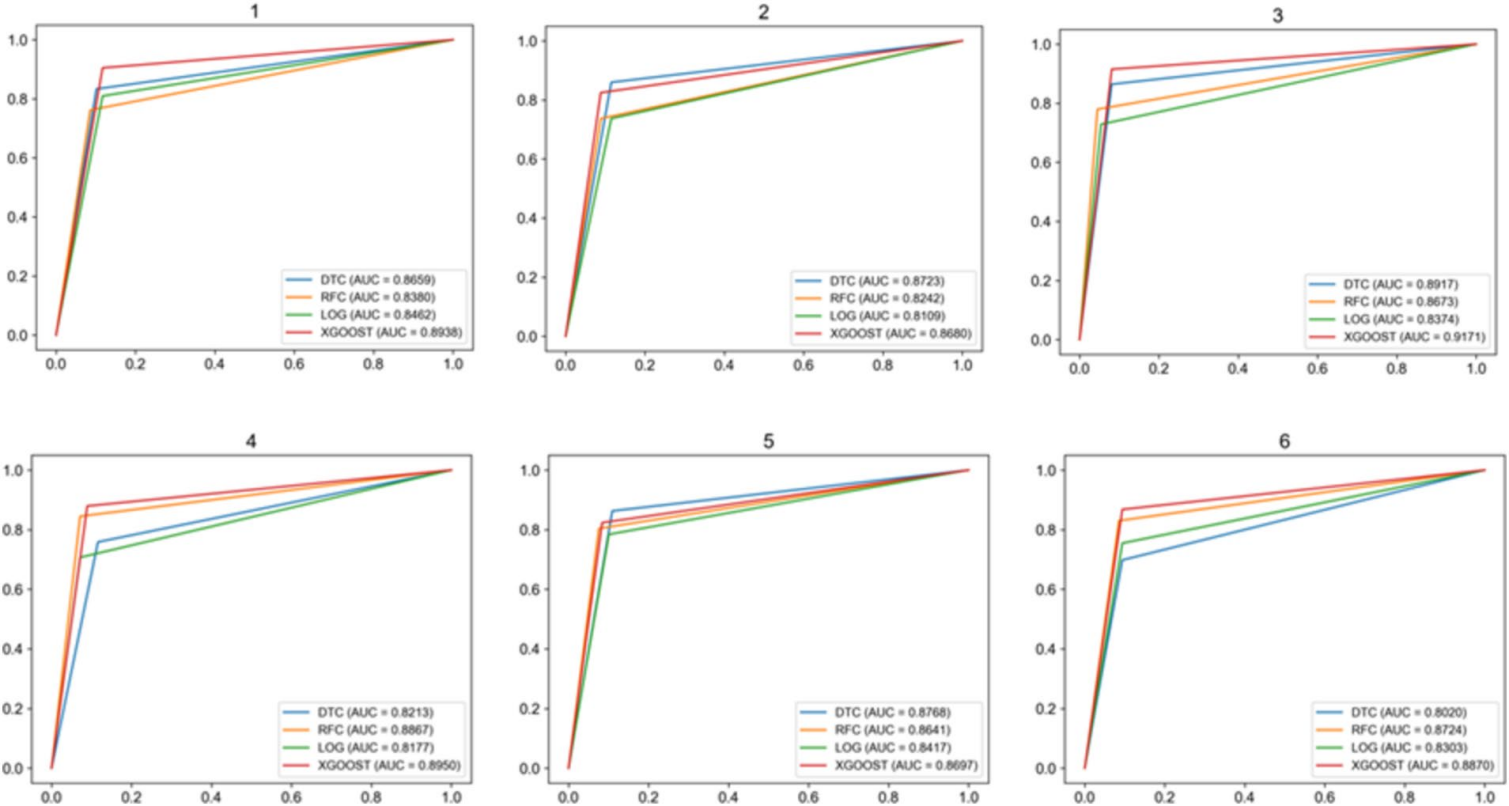
ROC curve.

The multiple randomized experiments of this study were designed to mitigate the overfitting or underfitting of the model on specific training and test sets to ensure the feasibility, stability, and reproducibility of the model. The confusion matrix is shown in Figure 3, and the recall, accuracy, and precision values are shown in Table 11. In Figure 4, the *ROC* curves were plotted based on the confusion matrix to assess the effectiveness of the model in predicting student academic achievement.

After six random experiments, we obtained the performance indicators such as confusion matrix as shown in Figure 3, the values of recall, accuracy and precision as shown in Table 12. The comprehensive average results show that the XGBoost model performs best in terms of recall and accuracy, reaching 86.52 and 89.22%, respectively. In addition, the ROC curve AUC (Area under ROC Curve) of the XGBoost model is the highest, indicating that it is the best predictor of students’ academic performance.

**Table 12:** The model predicts the outcome

Evalution criterion	Models	1(%)	2(%)	3(%)	4(%)	5(%)	6(%)	Average(%)
Recall	DTC	88.24%	87.65%	90.00%	87.65%	88.24%	84.12%	87.65%
	RFC	87.65%	85.29%	89.41%	85.48%	88.82%	88.82%	87.58%
	LOG	86.47%	83.53%	87.06%	81.54%	86.47%	85.88%	85.16%
	Xboost	88.82%	88.24%	91.76%	88.24%	88.82%	89.41%	89.22%
Accuracy	DTC	83.33%	85.96%	86.44%	85.48%	86.27%	69.81%	82.88%
	RFC	76.19%	73.68%	77.97%	79.03%	80.39%	83.02%	78.38%
	LOG	80.95%	73.68%	72.88%	72.58%	78.43%	75.47%	75.67%
	Xboost	90.48%	82.46%	91.53%	85.48%	82.35%	86.79%	86.52%
Precision	DTC	72.92%	79.03%	85.00%	81.54%	77.19%	77.08%	78.79%
	RFC	74.42%	80.77%	90.20%	89.09%	82.00%	81.48%	82.99%
	LOG	69.39%	76.36%	87.76%	83.33%	76.92%	78.43%	78.70%
	Xboost	71.70%	82.46%	85.71%	82.81%	80.77%	80.70%	80.69%

### 5.4 Learning data analysis

The research findings provide a comprehensive perspective on the factors influencing academic performance. The XGBoost model demonstrated excellent performance in terms of recall and accuracy, indicating its suitability for predictive analytics in educational settings. Key predictive factors include metacognitive awareness, learning motivation, engagement, time management, environmental factors, and mental health, all of which highlight the multidimensional nature of academic success. Furthermore, specialized training appears to bridge the gap between theoretical learning and practical skills, thereby enhancing students’ overall competencies.

Learning motivation plays a crucial role in students’ academic achievement. Psychology posits that needs are the intrinsic driving forces guiding all behaviors. When these needs translate into learning motivation, they become the driving force behind students’ continued learning and sustained behavior. Additionally, setting learning goals, appreciating academic content, and planning the learning process are key factors in forming strong learning motivation among students. Our findings validate this perspective, indicating that these factors significantly contribute to enhancing students’ academic performance.

However, in real life, some college students view obtaining a bachelor’s degree as their sole objective, lacking strong learning motivation, which leads to a more scattered approach to their daily studies. For instance, some students choose to indulge in entertainment activities when there are no class schedules, lacking effective study plans and goals. This lack of motivation and planning in their study attitudes makes it difficult for them to fully engage in academic tasks, subsequently affecting their academic performance. To improve academic outcomes, students need to cultivate intrinsic learning motivation, set clear academic goals, and strengthen self-management and time planning. Only by doing so can they achieve better success academically.

In addition to learning motivation, learning engagement is also one of the effective factors influencing academic performance. Learning engagement encompasses the level of student participation both inside and outside the classroom, such as in-class activities, extracurricular learning, and group discussions. Research has shown that high levels of learning engagement are often associated with better academic performance. Upon further investigation into students’ learning engagement, we found that factors such as the frequency of reviewing materials, asking teachers for help, the quality of homework completion, and attention in class can further reveal the specific impact of learning engagement on academic performance. Therefore, enhancing students’ learning engagement is an important pathway to improving academic results.

However, many students rely on last-minute cramming just before exams. While this “cramming” approach may yield short-term results in certain subjects, it may not be effective in others. This is because some subjects require long-term accumulation and deep understanding of knowledge, and relying solely on cramming does not allow for true mastery and application of the material. A long-standing lack of sustained learning engagement and participation may leave students with gaps in their knowledge acquisition and application, ultimately affecting their overall academic performance. Therefore, students should cultivate consistent learning engagement and balance daily study with last-minute review to ensure they can maintain excellent performance across all subjects.

Additionally, studies have indicated that the burden of academic tasks and the choice of learning methods significantly impact students’ grades. Faced with heavy academic pressure, students’ motivation to learn can be influenced by the complexity and quantity of tasks; an excessive workload may lead to frustration and hinder academic progress. Inappropriate learning methods can also result in insufficient mastery of knowledge, making it difficult to meet course requirements, thereby affecting academic performance. Therefore, providing effective learning support and methodological guidance to help students plan their academic tasks reasonably and improve learning efficiency is crucial for enhancing academic performance.

For example, courses such as university-level physics and organic chemistry, due to their complexity and challenges, often leave many students struggling to master effective learning methods. These courses not only require a solid foundation of knowledge but also demand that students be able to apply learning strategies flexibly, such as effective time management, phased learning, and techniques for consolidating knowledge. If students fail to master these methods, they may feel confused and frustrated, which can negatively impact their academic performance. Thus, helping students find learning methods that suit them, especially when facing challenging courses, is of utmost importance.

The research further found that specialized training has a positive impact on improving students’ academic performance. By combining theoretical knowledge with practical application, specialized training not only provides practical experience but also enhances students’ application abilities and skill levels, thereby effectively improving their academic performance. The ability to integrate theory and practice is of great significance for enhancing students’ overall quality.

Therefore, in educational management and guidance, in addition to optimizing the design and allocation of learning tasks, it is essential to focus on cultivating students’ learning methods and guiding them to set clear future goals. By providing diversified learning support and motivational measures, schools and educators can help students better cope with academic challenges and improve their academic performance. We hope this study can foster a positive learning attitude among students, assist them in enhancing their academic performance, and encourage teachers and educational institutions to play a more proactive role in providing comprehensive and personalized support for students. In this way, students can develop stronger self-directed learning abilities and practical experience, contributing more to the future development of society.

### 5.5 The limits and challenges of the research

It is known that overfitting is quite common in complex machine learning models, especially deep neural networks. Therefore, we implemented several measures in our research design to mitigate the effects of overfitting and ensure more stable performance of the model during both training and testing phases. Firstly, since our study is based on a relatively small dataset, we employed stratified sampling to ensure that the proportions of “passed” and “not passed” samples in the training and testing datasets are consistent with those in the original data. This approach helps to reduce the model’s excessive reliance on specific class samples and contributes to improving the model’s generalization ability. Secondly, we selected a feature set with fewer features to reduce model complexity and avoid overfitting in the feature space. The features selected through chi-squared tests ensured that each input feature is statistically significant, thus reducing the number of irrelevant features the model needs to learn, which lowers the complexity of the model. Finally, we designed a multi-round stratified sampling scheme, employing 5 rounds of random sampling during the construction of the training and testing sets. This method allows the model to learn multiple times from different sample distributions, further enhancing the model’s robustness (Li et al., 2022; Tsai et al., 2023). It is believed that the combination of these measures effectively reduces the risk of overfitting, thereby improving the model’s generalization ability and robustness.

However, the small sample size may not accurately reflect academic performance across various fields. Meanwhile, this study did not consider general education courses, such as College English, College Mathematics, and Moral Education, which limits the generalizability of the findings. Previous research indicates that parenting styles significantly affect children’s cognitive development, school readiness, and academic performance (Tripon, 2024). For example, students raised in democratic households typically exhibit open communication, supportive autonomy, and collaborative decision-making, which are linked to higher levels of school engagement and academic achievement. However, our study has not fully explored the impact of different parenting styles—such as authoritarian versus permissive democratic parenting—on college students’ academic success, particularly concerning their cognitive and social engagement.

While machine learning methods demonstrate some effectiveness, they need to be tailored to specific teaching contexts (Huang and Fang, 2013; Zhang and Dai., 2024). Future research should delve into additional factors affecting learning outcomes, such as social environments, family background differences, and cultural variations. Developing a more comprehensive scale for learning behavior characteristics is necessary to create a closed loop for textbook development in teaching experiments, research, and practice. Unfortunately, our research has not adequately addressed the differences in students’ family backgrounds, economic status, and cultural contexts, all of which may significantly influence their understanding of cognitive and social engagement, particularly in relation to the Sustainable Development Goals (SDGs) (Tripon, 2024). Meanwhile, it is crucial to further investigate the relationship between educators’ social–emotional behaviors and the well-being of college students, as well as how these factors contribute to achieving sustainable development goals. Accurately assessing the impact of democratic teaching on student achievements and social–emotional behaviors is essential for realizing long-term social benefits and aligning with multiple SDGs, such as SDG 3 (ensuring healthy lives) and SDG 16 (promoting peaceful and inclusive societies for sustainable development) (Tripon, 2024). Additionally, this study utilized only five machine learning models (LOG, SVC, RFC, XGBoost). The future education model can integrate more data analysis and assembling a variety of machine learning technologies to identify students’ learning motivations, mental health issues, improving the efficiency and effectiveness of academic performance and student engagement.

## 6 Conclusion

It is concluded that the factors affecting the academic performance of our students mainly involve metacognitive awareness, learning motivation, learning participation, environmental factors, time management and mental health. Metacognitive awareness includes factors such as college entrance examination scores, satisfaction and self-achievement. Learning motivation covers learning interest and other aspects. Learning engagement involves the extent to which students participate in classroom activities, extracurricular learning, and group discussions. High learning engagement is generally associated with better academic performance.

Although this study has improved the model’s generalization ability through methods such as stratified sampling, feature selection, and cross-validation, the model may experience a decline in performance when faced with new data due to the small dataset and fixed sample distribution. We recognize that overfitting is a common challenge in machine learning, particularly pronounced in complex models. Future research will focus on addressing these issues, including enhancing data diversity, conducting cross-domain validation, and employing model ensemble techniques to further improve the model’s generalization ability. Besides, more variables like time management, environmental factors and mental health, including classroom learning, self-learning, rational allocation of leisure time, home learning environment, library resources, laboratory facilities should be considered. These contents will contribute to a deeper understanding of the factors affecting students’ academic performance and provide guidance for the development of effective educational intervention strategies.

## Data Availability

The raw data supporting the conclusions of this article will be made available by the authors, without undue reservation.

## Footnotes

^1^ https://jiaowu.hebau.edu.cn/jxyj/rcpyfa.htm
